# Pepsin in the Treatment of Exposed Pulps

**Published:** 1873-03

**Authors:** 


*Pepsin in the Treatment of Exposed Pulps.*—Mr. Okley Coles, at a meeting of the Odontological Society of Great Britain, recommended the use of Pepsin in the treatment of Exposed Pulps. He says: "Early in the present year, being anxious to find an agent for the treatment of exposed pulp less dangerous in its character than arsenic, and at the same time less painful and fatal to the entire nerve, it occurred to me to try pepsin. My experiments, extending now to nearly a year, justify me in bringing it before the profession, with a view to a fuller trial of its merits and a larger field for its usefulness, if found as satisfactory in the hands of others as it has been in my own experience. The powdered pepsin (prepared by Messrs. Bullock & Reynolds) is mixed into a paste with dilute hydrochloric acid; *i. e.* with the dilute hydrochloric acid of the Pharmacopœia, diluted with 100 parts of water. This paste is left in contact with the pulp and covered with wax for three days; upon removing it the cavity is well washed out with warm water, and swabbed with carbolic acid dissolved in glycerine. The pulp is then capped and the cavity temporarily filled for some months, after which it is filled permanently. The use of the acid is to increase the action of the pepsin, while the glycerine is added simply to preserve the compound in the condition of a paste. Believing, as I do, in the advantage of preserving the healthy portion of a dental pulp, I have found pepsin an exceedingly valuable agent, since it acts only on that part of the pulp which is dead, or in an advanced stage of disintegration, (as the result of severe inflammation.) The fact that the pepsin paste produces no pain in digesting away the disorganized pulp tissue is, in most instances, a still further recommendation for its use."

